# Molekulare Laryngologie: Ein neues Kapitel im Verständnis laryngealer Erkrankungen

**DOI:** 10.1007/s00106-021-01016-1

**Published:** 2021-04-06

**Authors:** M. Gugatschka, T. Grossmann, D. Hortobagyi

**Affiliations:** Klinische Abteilung für Phoniatrie, Hals-Nasen-Ohren-Universitätsklinik, Medizinische Universität Graz, Auenbruggerplatz 26, 8036 Graz, Österreich

**Keywords:** Fibroblasten, Bioreaktoren, Stimmlippen, M. vocalis, Larynxschleimhaut, Fibroblasts, Bioreactors, Vocal cords, Vocal muscle, Laryngeal mucosa

## Abstract

**Hintergrund:**

Trotz erheblicher Fortschritte in der laryngologischen Forschung gibt es eine Reihe von (benignen) Stimmlippenerkrankungen, die kausal nicht therapierbar sind. Das liegt an der eingeschränkten Zugänglichkeit sowie der sensiblen Mikroarchitektur der Stimmlippen, die nicht auf zellulärer Ebene erforscht werden können. Das pathophysiologische Verständnis endet dadurch häufig in der makroskopischen Ebene, die Folgen von Interventionen werden großteils endoskopisch oder mit indirekten Methoden evaluiert.

**Fragestellung:**

Im nachfolgenden Beitrag stellen die Autor(inn)en biotechnologische State-of-the-Art-Methoden vor, die in der laryngologischen Forschung Anwendung finden, verbunden mit praktischen Beispielen.

**Ergebnisse:**

Tierversuche und Zellkulturexperimente haben in den letzten Jahren zu einer signifikanten Wissenserweiterung beigetragen, dies insbesondere in den Bereichen Stimmlippeninflammation und -narbenbildung. Dem Stimmlippenfibroblasten, als wichtigstem zellulärem Bestandteil der Lamina propria, kommt dabei eine zentrale Rolle zu.

**Schlussfolgerungen:**

Mittlerweile besteht bei einigen Krankheitsbildern ein tieferes Verständnis von Makroanatomie und Makropathophysiologie als je zuvor. In-vitro-Versuche zeigten beispielsweise, dass Stimmlippenfibroblasten in einem inflammatorischen Setting weniger profibrotische und proinflammatorische Zytokine sezernierten, wenn sie Vibrationen ausgesetzt sind. Umgesetzt auf die Klinik könnte das bedeuten, dass eine frühe Stimmaktivierung nach operativen Eingriffen an den Stimmlippen zu besserer Heilung und besseren stimmlichen Ergebnissen führt. Unsere Vision lautet, dass die molekulare Laryngologie ein gesichertes Fundament an Wissen bereitstellen soll, auf das in weiterer Folge klinische Studien aufgebaut werden können.

## Abstract (en)

**Background:**

Despite considerable advances in laryngological research, there is still a plethora of (benign) vocal fold pathologies that cannot be treated causally. This is due to the limited accessibility and sensitive microarchitecture of the vocal folds, which cannot be investigated at a cellular level. Consequently, current pathophysiological knowledge is frequently based on macroscopic findings. The impact of interventions is mainly evaluated endoscopically or via indirect diagnostic methods.

**Objective:**

The aim of this article is to discuss state-of-the-art biotechnological methods used in laryngological research, illustrated by practical examples.

**Results:**

In recent years, animal and in vitro experiments have significantly contributed to a continuous expansion of knowledge in this field, particularly regarding vocal fold inflammation and scar formation. Vocal fold fibroblasts, the most important cellular component of the lamina propria, can be accredited a central role in these processes.

**Conclusion:**

Our knowledge regarding macroanatomy and macropathophysiology of several pathologies has increased considerably in recent years. In vitro trials have shown, e.g., that vocal fold fibroblasts in an inflammatory setting secrete less profibrotic and proinflammatory cytokines when exposed to vibration. Early vocal exercises after surgical interventions on the vocal folds may therefore promote better wound healing and consequently improved phonation. Research in molecular laryngology should create a solid basis of knowledge for subsequent clinical studies.

Die Stimmlippen (SL) des Menschen sind einzigartigen mechanischen Belastungen ausgesetzt. Bei Phonation treten Beschleunigungen von 200–300 G und Frequenzen von 100–1000 Hz auf [1]. Um diesen Belastungen standzuhalten und zudem eine ungestörte SL-Schwingung mit daraus resultierendem klaren Stimmklang zu gewährleisten, verfügt das ortsständige Gewebe über Eigenschaften, wie man sie nirgendwo im Körper findet.

Die laryngeale Mukosa spielt eine herausragende Bedeutung für die Stimmproduktion

Insbesondere die laryngeale Mukosa spielt eine herausragende Bedeutung für die Stimmproduktion, was sich eindrucksvoll im Fall einer ein- oder beidseitigen SL- Narbe (bei der die Schwingung stark eingeschränkt ist) akustisch manifestiert. Im folgenden Beitrag wird die bekannte und vielfach beschriebene SL-Anatomie um neue Aspekte ergänzt. Darauf aufbauend gehen die Autor(inn)en auf benigne Veränderungen der SL-Mukosa, insbesondere der funktionell wichtigen Lamina propria, näher ein, wohl wissend, dass viele Erkrankungen vom SL-Epithel (v. a. Karzinome, Papillome) und der SL-Muskulatur ausgehen (beispielsweise altersassoziierte Atrophie) bzw. neurogener Natur (Parese) sind.

Benigne Veränderungen der Lamina propria kommen sehr häufig in der phoniatrisch-laryngologischen Praxis vor und können entweder konservativ-logopädisch oder (mikro)chirurgisch behandelt werden. Die Diagnose und phonochirurgische Therapie einer SL-Zyste oder eines SL-Polypen stellen in den meisten Fällen kein Problem dar. Es gibt aber auch eine Reihe von benignen SL-Veränderungen, bei denen eine chirurgische Therapie zwar zu einer Verbesserung der Symptome, aber keiner Restitutio ad integrum führt. Beispiele hierfür sind die zuvor genannten SL-Narben, aber auch ein Sulcus glottidis oder in manchen Fällen das Reinke-Ödem.

## Herausforderungen in der Stimmlippenforschung

An dieser Feststellung offenbart sich ein fundamentales Problem in der Laryngologie: Das pathologische/pathophysiologische Verständnis endet häufig in der mikroskopischen, bisweilen sogar in der makroskopischen Ebene, wie im Fall des Reinke-Ödems, bei dem eine Blickdiagnose in den allermeisten Fällen zwar ausreichend ist, die genauen Krankheitsmechanismen aber im Dunkeln liegen. Die zellulären Prozesse, die in den SL nach einer Verletzung (Intubation, Operationen), durch Nikotinabusus oder Stimmmissbrauch ablaufen, sind beim Menschen beispielsweise weitgehend unbekannt.

Ein Grund für die unzureichende Wissenslage besteht in der besonderen anatomischen Lage und Struktur der SL(‑Mukosa) selbst: Die funktionell wichtige Pars membranacea (der „schwingende Teil“) ist nur etwa 1,6 bzw. 1,2 cm lang (beim Mann bzw. bei der Frau) und kann aus ethischen Gründen nicht für wissenschaftliche Fragestellungen biopsiert werden, da jede Biopsie das Risiko einer permanenten Schleimhautvernarbung in sich trägt. Ebenso sind Nachkontrollen nach Interventionen auf lupenlaryngoskopische Untersuchungen oder auf indirekte Parameter, wie Stimmanalysen oder subjektive Scores (z. B. Voice Handicap Index, VHI; Stimmstörungsindex, SSI; usw.), begrenzt.

Die dargestellten Schwierigkeiten führten dazu, dass wichtige Fragen der SL-Biologie und zugehörigen Pathophysiologie unerforscht sind. Somit bleibt eine Reihe von fundamentalen physiologischen und pathophysiologischen Fragen offen, wie etwa die Interaktionen Epithel – Lamina propria, die Auswirkungen von Allergien auf die SL, die Rolle von Stimmruhe/-aktivierung nach SL-Operationen, der Einfluss von Hormonen u.v.a.m. In letzter Konsequenz bedeutet dies aber auch, dass einige benigne Erkrankungen der SL nicht kausal therapierbar sind.

Es gibt nur wenige Arbeitsgruppen, die sich mit molekularen Fragestellungen in menschlichen SL befassten, darunter Verdolini et al., die Sekretabstriche von SL nach Stimmbelastung durchführten und auswerteten [2]. Sie wiesen im Sekret erhöhte Spiegel inflammatorischer Zytokine nach. Gleichzeitig betonen die Autoren auch die Schwierigkeit in der Methode der Probengewinnung, was sich auch in der niedrigen Probandenzahl niederschlug (*n* = 3). Die SL der Probanden mussten oberflächenanästhesiert werden, was zudem zu einer Verdünnung des gewonnenen Sekrets führte und die Auswertung erschwerte. Gleichzeitig kann aus dem gewonnenen Sekret nicht die Frage geklärt werden, ob die freigesetzten Zytokine vom Epithel oder der Lamina propria ausgingen.

## Alternative Modelle

Alternativen, sich diesen Fragestellungen anzunähern, sind Zellkulturmodelle und Tiermodelle. Bereits in den frühen 2000er-Jahren gab es eine Vielzahl an Publikationen in dem Bereich, aus welchen auch wichtige Erkenntnisse über Zellfunktionen gewonnen wurden [3–7].

Alternativen, sich diesen Fragestellungen anzunähern, sind Zellkulturmodelle und Tiermodelle

Einschränkend muss hier erwähnt werden, dass sich die Forschung hauptsächlich auf SL-Fibroblasten, als wichtigstem Zelltyp der Lamina propria fokussierten. Der Grund, warum das humane SL-Epithel schlechter erforscht ist, liegt in der Tatsache begründet, dass es ungleich schwieriger ist, dieses zu gewinnen, zu isolieren und zu kultivieren. Bis dato gibt es auch nur wenige Arbeiten, die sich mit dem Grenzgebiet zwischen Lamina propria und Epithel beschäftigen [8, 9].

Eine Vielzahl von Studien befasste sich im Tiermodell (meistens Ratten) mit Inflammationsprozessen nach chirurgisch induzierten Verletzungen [6, 10–14]. Obwohl diese Prozesse teilweise gut erforscht sind, gibt es darauf aufbauend jedoch keine Therapien für Anwendungen beim Menschen. Prinzipiell geht das Bestreben in den letzten Jahren hin zur Vermeidung von Tierversuchen.

## Mikroanatomie und Mikrophysiologie

**Figure 1:**
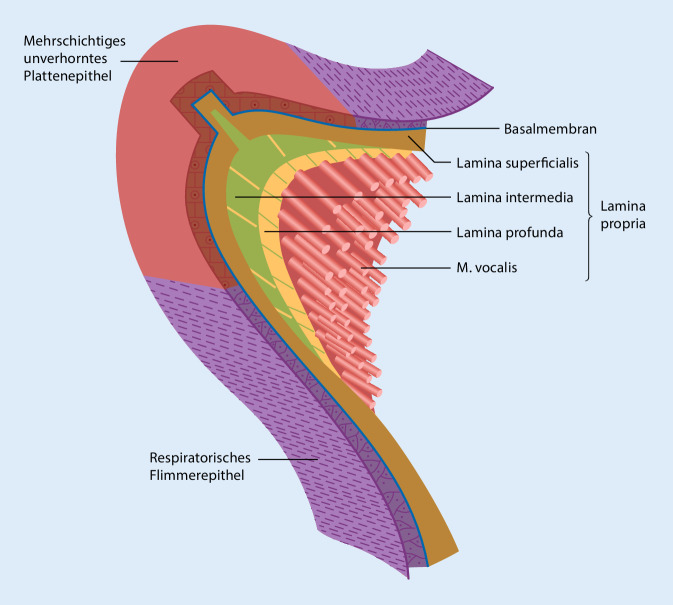

Abseits der bekannten Literatur zur SL-Anatomie gehen die Autor(inn)en auf weniger bekannte Aspekte in der Mikroanatomie ein, die funktionell eine wichtige Rolle spielen. Danach werden einige bekannte Krankheitsbilder (Reinke-Ödem, SL-Narbe) erörtert und diese unter dem Blickwinkel molekularer Mechanismen neu betrachtet (Abb. 1).

Unter dem Begriff der SL-Mukosa, also der Schleimhaut, werden das Epithel, die Basalmembran und alle 3 Schichten der Lamina propria zusammengefasst [15, 16]. Diese liegt dem M. vocalis als oberflächlichem Anteil des M. thyroarytaenoideus auf. Minoru Hirano publizierte 1974 erstmals die mittlerweile berühmte Body-Cover-Theorie der SL-Schwingung [17]. Dabei beschrieb er den einzigartigen morphologischen Aufbau der SL und entwickelte ein biomechanisches Modell, indem er sie funktionell in „body“ (M. vocalis und Lig. vocale) und „cover“ (Lamina superficialis, Basalmembran und Epithel) einteilte und darin die SL-Funktion als „double-structured vibrator“ beschrieb.

### Epithel

**Figure 2:**
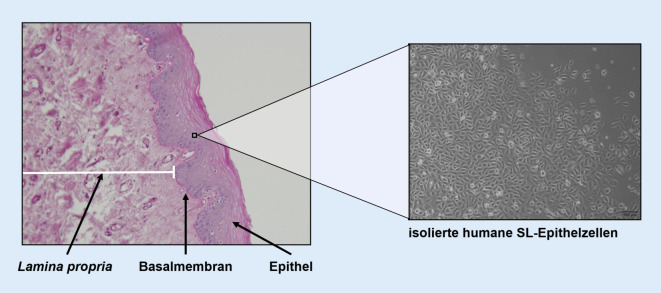

Im Gegensatz zum einschichtigen respiratorischen Epithel des restlichen Atemwegs sind die SL von einem mehrschichtigen unverhornten Plattenepithel überzogen, das über interzelluläre Kontakte eng miteinander verbunden ist, damit es der mechanischen Belastung während der Phonation standhält (Abb. 2). Die Integrität der Zellverbindungen ist zudem auch für die Absorption und Sekretion von Ionen und somit für die Zusammensetzung der epithelbedeckenden Schleimschicht unverzichtbar. Dieser Schleim ist eine wichtige Barriere gegen biologische und chemische Noxen und spielt eine wichtige Rolle in der Befeuchtung der SL.

Die Furchen und Falten der luminalen Oberfläche des Epithels erleichtern die Randkantenverschiebung

Bei der mikroskopischen Betrachtung der luminalen Oberfläche des Epithels fällt auf, dass diese nicht, wie die makroskopische Untersuchung vermuten würde, völlig glatt ist, sondern viele kleine Furchen und Falten aufweist. Diese erleichtern einerseits die Randkantenverschiebung während der Stimmbildung und ermöglichen andererseits eine bessere Haftung des Schleimteppichs [18–20].

### Basalmembran

Relativ wenig Beachtung findet die Basalmembran, der aber als stabilisierender Struktur zwischen SL-Epithel und darunter liegender Lamina propria eine wichtige Rolle zukommt. „Anchoring fibers“ (bestehend aus Kollagen Typ IV) sorgen für eine Verankerung zwischen Epithel und Lamina propria. Die Anzahl dieser „anchoring fibers“ ist abhängig von der Lokalisation, so ist sie in der Pars membranacea höher als an anderen Stellen [15]. Gray, der als Erstbeschreiber dieser Fasern gilt, postulierte zudem einen genetischen Einfluss auf die Faserdichte, was unterschiedliche Anfälligkeiten (beispielsweise für organische SL-Veränderungen) auf Vibrationsstress erklären könnte.

### Lamina propria

**Figure 3:**
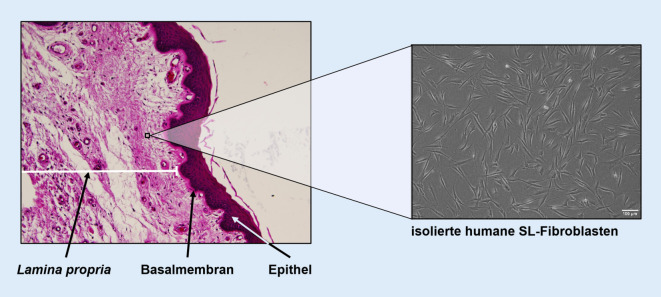

Der schichtweise Aufbau der SL-Mukosa spielt funktionell eine wesentliche Rolle und besteht aus zellulären und extrazellulären Komponenten. Bekannterweise besteht die menschliche Lamina propria aus 3 Schichten (Lamina superficialis – intermedia – profunda), wobei die „Festigkeit“ von luminal nach basal graduell zunimmt. Der wichtigste zelluläre Bestandteil ist der SL-Fibroblast (SLF; Abb. 3). Dieser spielt eine zentrale Rolle in der Produktion von Extrazellulärmatrix(ECM)-Bestandteilen wie Hyaluronsäure, verschiedenen Typen von Kollagenen, elastischen Fasern, Fibronectin u.v.a.m. Die Schwingungseigenschaften gesunder SL hängen entscheidend von der Homöostase der ECM-Bestandteile und dem Gleichgewicht zwischen Synthese und Degradation ab. Einer erheblichen Zugbelastung während der Phonation sind insbesondere das vordere und hintere Ende der SL, die Maculae flavae, ausgesetzt. Ebenda befindet sich ein spezieller Subtyp der SLF, die sog. sternförmigen Zellen („stellatae cells“) [21, 22]. Unter diesen befindet sich ein hoher Anteil an Stammzellen, die nach einer SL-Verletzung aktiviert werden und migrieren sowie an der Entzündungsreaktion beteiligt sind [23]. Bei Säuglingen ist die Dreischichtigkeit der Lamina propria noch nicht ausgebildet. Bisher ist noch nicht endgültig geklärt, wie die unterschiedliche Zusammensetzung der ECM entsteht, eine regelmäßige Phonation scheint jedoch eine wesentliche Rolle zu spielen. Es wird vermutet, dass die Kräfte während der Stimmbildung je nach Lokalisation unterschiedlich stark auf die SLF einwirken. Diese Reize bewirken, dass beispielsweise Kollagen in unterschiedlichen Konzentrationen produziert wird [24].

### M. vocalis

Die tiefste Schicht der SL bildet der M. vocalis. Er wird als oberes, freies Ende des M. thyroarytaenoideus angesehen. Bei genauerer histologischer Betrachtung fällt eine besonders hohe Dichte an Muskelspindeln auf. Diese verfügen über spezielle Rezeptoren, die die aktuelle Dehnung des Muskels erfassen. Das ist ein deutlicher Hinweis dafür, dass er nicht nur als statisches Fundament zu betrachten ist, sondern für die Feinregulation der SL-Spannung und somit der Tonhöhe mitverantwortlich ist [25, 26].

## Mikrophysiologie/Pathophysiologie

Der Wissensstand über die zellulären Vorgänge nach einer SL-Verletzung (vornehmlich die SLF betreffend) im Tiermodell ist gut. Einschränkend muss erwähnt werden, dass oft eine genauere Differenzierung der Inflammation nicht möglich ist, d. h., es ist nicht zuordenbar, ob die Herauf‑/Herunterregulationen von bestimmten Zytokinen auf SL-Epithelzellen, SL-Fibroblasten oder ortsständige Makrophagen zurückzuführen sind.

Mikroanatomie und Mikrophysiologie sind die Schlüssel im Verständnis zur molekularen Laryngologie. Welham et al. zeigten im Tierversuch, dass es eine bzw. 4 h nach einer SL-Verletzung zu einem Anstieg bestimmter proinflammatorischer und profibrotischer Faktoren kommt [10]. Dazu zählen unter anderem Cyclooxygenase‑2 (COX-2), Tumornekrosefaktor-alpha (TNF-α), Interleukin-1beta (IL-1β). Einige dieser Faktoren decken sich mit jenen aus der zuvor zitierten Humanstudie, bei der nach Stimmbelastung Abstriche von den SL gewonnen wurden [2]. Es erscheint auch plausibel, dass sich Faktoren nach SL-Verletzung (beispielsweise Operationen, Intubation) und Stimmbelastung zwar quantitativ, aber nicht qualitativ unterscheiden.

Besonders interessant erscheint die Fragestellung, warum manche SL-Verletzungen folgenlos ausheilen, während andere mit einer Narbenbildung einhergehen. Zwar ist die Wahrscheinlichkeit naturgemäß von der Größe der Läsion/Resektion abhängig, es spielen aber auch andere Faktoren eine Rolle. Es ist bekannt, dass sich nach einer Verletzung SL-Fibroblasten in sog. Myofibroblasten oder Narbenfibroblasten differenzieren, die ein anderes Profil an Genen und Proteinen exprimieren. Dass von diesen Zellen u. a. mehr Kollagen und weniger Hyaluronsäure produziert wird, ist das morphologische Korrelat der SL-Narbe [27]. Ergebnisse der In-vitro-Versuche der Autor(inn)en zeigten, dass eine unmittelbare mechanische Aktivierung nach inflammatorischem Stimulus zu einer niedrigeren Expression inflammatorischer und fibrotischer Gene führten [28]. Auch Geschlechtsunterschiede in der ECM-Komposition stehen im Verdacht, Auswirkungen auf die Häufigkeit von SL-Veränderungen zu haben: Die Lamina propria von Männern weist mehr Hyaluronsäure als die von Frauen auf, wodurch ein stärkerer Schutz vor Phonotraumen ausgehen könnte [29]. Dies erklärt nach Meinung der Autoren die niedrigere Prävalenz von Phonotraumata bei Männern im Vergleich zu Frauen.

## Spezifische Krankheitsbilder

### Reinke-Ödem

Das Reinke-Ödem ist in den allermeisten Fällen eine Blickdiagnose in der phoniatrisch-laryngologischen Praxis. Typischerweise sind Frauen um das 50. Lebensjahr betroffen, die zudem eine Raucheranamnese aufweisen und häufig (berufliche) Vielsprecher sind. Das lupenlaryngoskopische Bild ist gekennzeichnet durch voluminöse SL mit ektatischen und varikösen Blutgefäßen, die durch die Mukosa durchschimmern. Die Anamnese führte zur Annahme, dass sowohl Nikotinabusus als auch hormonelle Ursachen an der Pathogenese beteiligt sein könnten. Bei genauer Betrachtung zeigt sich jedoch, dass viele Annahmen nicht evidenz-, sondern nur fallbasiert sind, weswegen viele Fragen offenbleiben: Was ist beispielsweise die genaue Rolle der (Geschlechts‑)Hormone in der Entstehung des Reinke-Ödems, und welche Folgen hat eine Hormonersatztherapie auf die Stimme? Gibt es eine Überrepräsentation von Frauen, nur weil diese eher die Sprechstunde aufsuchen und eine tiefere Stimme Männer nicht so sehr stört (und sogar als angenehm, weil sehr männlich, empfunden wird)?

Die meisten Studien berichten von einer Überrepräsentation von weiblichen Patientinnen, es gibt aber auch Ausnahmen wie Kravos et al., die ein großes Kollektiv von 56 Männern mit Reinke-Ödem beschreiben [30]. Der Einfluss von Zigarettenrauch gilt als gesichert, selbst wenn die Pathomechanismen bisher unklar sind. Schwierig wird es, die einzelnen additiven Effekte der Konoxen zu quantifizieren: Wie ist die Rolle des laryngopharyngealen Refluxes (LPR) einzuschätzen, der von einigen Autoren als „erwiesen“ angesehen wird, von anderen aber nicht [31]? Welchen zusätzlichen Einfluss hat die Stimmbelastung auf die Pathogenese?

Im Bioreaktor zeigte sich die vermehrte Expression einiger Gene nur bei Vibration *und* Zigarettenrauch

Das Wissen über die zellulären Mechanismen im Fall des Reinke-Ödems ist gering. Manche Autoren postulieren eine proangiogenetische Achse in der Pathogenese, dazu passen Beschreibungen von Sato et al., die in entsprechenden Histologiepräparaten VEGF („vascular endothelial growth factor“) in ortsständigen Makrophagen immunhistochemisch nachweisen konnten [4]. Elektronenmikroskopische Untersuchungen derselben Gruppe beschreiben fenestrierte und lückenhafte Gefäße (vibrationsbedingt?), die wohl Hauptursache für das Ödem sein dürften [4]. Duflo et al. führten Micro-Array-Analysen u. a. an Reinke-Ödem-Proben durch, jedoch fehlte es in der Studie an Kontrollproben, d. h. es wurden Reinke-Ödem-Proben mit Proben aus SL-Polypen verglichen [32]. In den Ergebnissen beschrieben sie, dass in Reinke-Ödem-Proben antioxidative Gene hochreguliert waren (was in Hinblick auf den oxidativen Stress, verursacht durch Nikotinabusus, nicht verwundert), aber sie erwähnten auch, dass kollagenassoziierte Gene vermindert exprimiert wurden.

Die eigenen Experimente der Autor(inn)en an humanen SLF bestätigen diese Ergebnisse, zusätzlich stellten die Autor(inn)en eine signifikante Hochregulation der Hyaluronsäureproduktion (womöglich als protektiver Mechanismus) fest [33]. Die Verringerung des Kollagengerüsts in der Lamina propria, gepaart mit mehr Hyaluronsäure, kann demzufolge als zusätzlicher Faktor in der Ödementstehung gesehen werden. Eine rezente Studie der Arbeitsgruppe der Autor(inn)en ergab unter Verwendung eines von der Arbeitsgruppe entwickelten phonomimetischen Bioreaktors, dass einige Gene im Zusammenhang mit dem Reinke-Ödem nur durch eine Kombination von Vibration *und* Zigarettenrauch vermehrt exprimiert wurden (v. a. gefäßaktive VEGF). Eine Stimulation mit nur einem der beiden führte zu keiner signifikanten Veränderung. Gleichzeitig wurden durch Zigarettenrauch getriggerte hohe COX-2-Spiegel durch zusätzliche Vibration herunterreguliert [34].

### Stimmlippennarben

Ein- oder beidseitige SL-Narben gehen mit erheblichen Funktionseinbußen einher, die sich klinisch als Heiserkeit und rasche Stimmermüdung manifestieren. SL-Narben gehen, wie erwähnt, auf mikroskopischer Ebene mit einer Differenzierung der SLF in sog. Myofibroblasten/Narbenfibroblasten einher. Diese unterscheiden sich nicht nur phänotypisch von normalen SLF, sondern produzieren auch ein anderes Muster an ECM-Bestandteilen.

Als Konsequenz findet sich in vernarbten SL überschüssiges Kollagen, das nicht mehr parallel zum freien SL-Rand liegt, sowie reduzierte Anteile an Elastin und Hyaluronsäure. Dies führt zu einer Verschlechterung der viskoelastischen Eigenschaften und in weiterer Folge zu einem unregelmäßigen Schwingungsablauf während der Phonation. Offenbar spielt das Alter, in dem eine Verletzung auftritt, eine wichtige Rolle, wie die eigenen Versuche der Autor(inn)en zeigten. Myofibroblasten jüngerer Versuchstiere produzierten auch 3 Monate nach initialem Trauma höhere (narbenprotektive) Hyaluronsäurespiegel als mittelalte Tiere [35]. Gleichzeitig sprechen die Myofibroblasten jüngerer Tiere stärker auf das antifibrotische Zytokin „hepatocyte growth factor“ (HGF) an als die Zellen älterer Tiere [36].

In vernarbten SL findet sich überschüssiges Kollagen, das nicht mehr parallel zum freien SL-Rand liegt

In den letzten Jahren gab es immer wieder Versuche, bei denen verschiedene Arten von Stammzellen auch im humanen Setting in SL injiziert wurden. Die Ergebnisse waren, ebenso wie die Krankheitsbilder, sehr variabel und wurden nicht immer mit objektiven Parametern gemessen [37, 38]. Zudem stellt sich die prinzipielle Frage, ob Stammzellen es tatsächlich vermögen, die ursprüngliche Gewebsstruktur inkl. Schichtung der Lamina propria wiederherzustellen. Stammzellen produzieren eine Vielzahl von Wachstumsfaktoren und Zytokinen, und es bedarf einer sorgfältigen Risikoabschätzung, diese in SL zu injizieren. Wenn es nur darum geht, die SL-Narbe „weicher“ und somit schwingungsfähiger zu machen, wären einzelne Faktoren in Betracht zu ziehen (wie beispielsweise HGF) und nicht ein „Cocktail“ verschiedener Wirkstoffe in unbekannten Dosierungen, wie sie von Stammzellen sezerniert werden. Die Gruppe um D. Chhetri publizierte bereits 2011 eine Studie, in der durch die Injektion von autologen Fibroblasten in vernarbte SL eine Verbesserung der Schwingungseigenschaften eintrat [39]. Dieser Ansatz wurde jedoch in der Community nicht aufgegriffen, und es wurden, wie erwähnt, vermehrt Stammzellen für die Versuche herangezogen.

Insgesamt wären also auch im Bereich der SL-Narben kausale Therapieoptionen wünschenswert, beispielsweise ein vollständiger autologer Gewebsersatz, wie es die Gruppe um Nathan Welham ansatzweise publizierte [9]. Einen sehr innovativen, aber noch nicht umsetzbaren Ansatz publizierten Kishimoto et al., die sich das Prinzip des „genome editing“ zunutze machten. Dieses basiert auf dem punktgenauen und gezielten Herausschneiden und Ersetzen von Gensequenzen mithilfe von „Genscheren“. Im Rahmen ihrer Versuche induzierten sie SL-Narben bei Ratten und injizierten anschließend lokal einen siRNA(Small-Interfering-RNA)-Liposom-Komplex. Dieser Komplex soll die Expression von SERPINH‑1, eines Proteins, das für die Faltung von Kollagen verantwortlich ist, unterdrücken. Sie konnten zeigen, dass so die mit der Vernarbung assoziierte Kollagenakkumulation der SL signifikant geringer ausfiel und sich somit positiv auf die Wundheilung auswirkte [40].

## Ausblick

In den letzten Jahren hat sich das Wissen um zelluläre Prozesse in den SL erheblich erweitert. Es muss das langfristige Ziel sein, diese Erkenntnisse auch in den klinischen Alltag zu integrieren und auf der Basis des Wissens kausale Therapieformen zu etablieren. Dies sollte die Verwendung einzelner Faktoren, aber auch die Züchtung von Mukosatransplantaten beinhalten. Die Vision im ersten Fall könnte lauten, Substanzen zu entwickeln (beispielsweise im Fall des Reinke-Ödems), die dann „office-based“ in die SL injiziert werden könnten. Die Züchtung eines autologen Mukosatransplantats wäre ein Durchbruch in der Behandlung von SL-Narben. Auch die Entwicklung spezifischer biokompatibler Gele, die als Träger für Zellen und Wirkstoffe dienen, ist ein möglicher und klinisch gut vorstellbarer Weg. Die Aufgabe solcher Gele wäre es sicherzustellen, dass die Wirksubstanzen möglichst lange am Ort der Applikation bleiben.

Die Arbeitsgruppe der Autor(inn)en hat in den letzten Jahren Protokolle entwickelt, mit welchen sie Epithelzellen und Fibroblasten aus laryngealer Mukosa isolieren, charakterisieren und kultivieren kann [28, 33, 41]. Ein weiterer wesentlicher Fortschritt war die Entwicklung geeigneter Zellkulturapparate, die die SL-Vibrationen auch unter Kulturbedingungen applizieren können. Dies ist essenziell, da man weiß, dass SLF unter statischen Bedingungen, d. h. ohne Vibration, dedifferenzieren. Sie verlieren mit der Zeit ihre ortsspezifischen Qualitäten und entwickeln sich in Richtung „normaler“ Fibroblasten [1].

Weltweit gibt es etwa 6 verschiedene Prototypen für derartige „Bioreaktoren“

**Figure 4:**
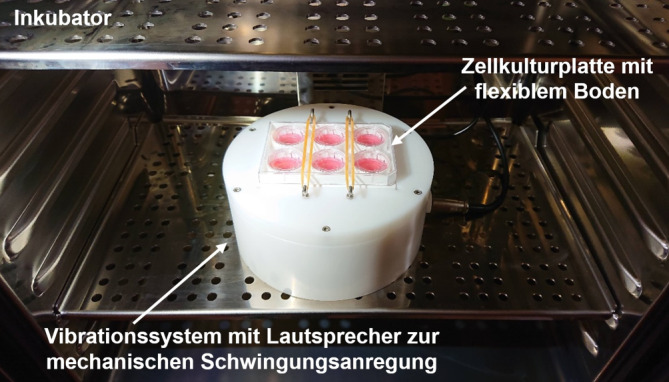

Weltweit gibt es etwa 6 verschiedene Prototypen für derartige „Bioreaktoren“, wobei keiner davon kommerziell erhältlich ist. Die Gruppe der Autor(inn)en hat in den letzten Jahren einen derartigen „phonomimetischen“ Bioreaktor entwickelt und führt damit bereits Experimente zu verschiedenen Fragstellungen durch [28, 34, 41]. Der Vorteil des Prototypen der Autor(inn)en besteht darin, dass er aus kommerziell erhältlichen Einzelteilen besteht und deswegen relativ leicht von anderen Gruppen nachgebaut werden kann. (Abb. 4) Zudem können die Autor(inn)en die kultivierten SLF mit jeder beliebigen Frequenz anregen, was das Gerät einzigartig macht. Derzeit laufen Bemühungen, diesen Bioreaktor auch für dynamische Kokulturen (Epithelzellen und Fibroblasten) im 3‑D-Setting zu etablieren. Ein solches organotypisches Modell wäre in vielen Eigenschaften mit nativer Larynxmukosa vergleichbar und könnte für eine Vielzahl physiologischer und pathophysiologischer Fragstellungen herangezogen werden. Zudem könnten derart Medikamente einfach und schnell in vitro getestet werden.

Zu den wichtigsten Arbeiten der letzten Jahre im Gebiet der SL-Biologie zählt zweifelsohne die zitierte Studie der Gruppe um Nathan Welham [9]. Die Gruppe kokultivierte humane Epithelzellen und SLF und stellte fest, dass sich nach 14 Tagen eine Basalmembran herausgebildet hatte. Die derart gezüchteten Mukosakonstrukte testeten sie histologisch, proteomisch, immunologisch und hinsichtlich mechanischer Belastbarkeit. Die Ergebnisse zeigten, dass die gezüchtete Mukosa in vielerlei Hinsicht große Ähnlichkeit mit nativer humaner Mukosa hatte, zudem ein niedriges immunologisches Profil aufwies und über bessere Schwingungseigenschaften verfügte als orale Mukosa. Die Arbeit stellt einen bedeutenden Schritt im Bereich des laryngealen Tissue-Engineerings dar und gibt berechtigten Grund zur Hoffnung, dass eines Tages autologe Mukosakonstrukte als Gewebsersatz zur Verfügung stehen könnten.

Auch pluripotente Stammzellen haben bereits Eingang in die SL-Forschung gefunden. Epithelzellen sind, wie erwähnt, ein schwer zu isolierender Zelltyp, weswegen die Gruppe um Susan Thibeault Stammzellen in Epithelzellen transformierte: Sie kultivierten iPS („induced pluripotent stem cells“) zusammen mit Fibroblasten und stellten fest, dass diese nach 4 Wochen auf genetischer Ebene ihre pluripotenten Merkmale verloren hatten und gleichzeitig für Epithelzellen spezifische Proteine (Keratin 13 und 14) produzierten. Auch diese Studie ist ein wichtiger Schritt in Richtung des autologen Gewebsersatzes [8].

## Fazit für die Praxis

- Die Schleimhaut der menschlichen Stimmlippen (SL) kann nicht für wissenschaftliche Fragestellungen biopsiert werden, da jede Biopsie das Risiko einer permanenten Schleimhautvernarbung in sich trägt.
- Das erschwert das pathophysiologische Verständnis der SL-Erkrankungen.
- Funktionell wichtig ist Pars membranacea, sie ist beim Mann etwa 1,6 und bei der Frau etwa 1,2 cm lang.
- Die Furchen und Falten der luminalen Oberfläche des Epithels erleichtern die Randkantenverschiebung
- Funktionseinbußen bei SL-Narben zeigen sich als Heiserkeit und rasche Stimmermüdung.
- In Narben differenzieren die SL-Fibroblasten in sog. Myofibroblasten/Narbenfibroblasten.
- Offenbar spielt das Alter, in dem eine Verletzung der SL auftritt, eine wichtige Rolle für die Regeneration.
- In Proben aus Reinke-Ödemen waren antioxidative Gene hochreguliert und kollagenassoziierte Gene vermindert exprimiert.
- Die Verringerung des Kollagengerüsts in der Lamina propria kann ein zusätzlicher Faktor in der Ödementstehung sein.
- Versuche mit unserem phonomimitischen Bioreaktor zeigen, dass Vibration im Anschluss an einen inflammatorischen Stimulus zu verringerter Expression von inflammatorischen und fibrotischen Zytokinen führt. Dies könnte ein Hinweis sein, dass frühe Stimmaktivierung im Anschluss an Stimmlippen-OPs den Heilungsverlauf positiv beeinflusst.
